# Feature Selection Model Based on IWOA for Behavior Identification of Chicken

**DOI:** 10.3390/s22166147

**Published:** 2022-08-17

**Authors:** Lihua Li, Mengzui Di, Hao Xue, Zixuan Zhou, Ziqi Wang

**Affiliations:** 1 College of Mechanical and Electrical Engineering, Hebei Agricultural University, Baoding 071000, China; 2 Key Laboratory of Broiler Layer Breeding Facilities Engineering, Ministry of Agriculture and Rural Affairs, Baoding 071000, China; 3 Hebei Provincial Key Laboratory of Livestock and Poultry Breeding Intelligent Equipment and New Energy Utilization, Baoding 071000, China

**Keywords:** breeding chickens, acceleration sensor, IWOA–XGBoost, feature optimization, behavior recognition

## Abstract

In order to reduce the influence of redundant features on the performance of the model in the process of accelerometer behavior recognition, and to improve the recognition accuracy of the model, this paper proposes an improved Whale Optimization algorithm with mixed strategy (IWOA) combined with the extreme gradient boosting algorithm (XGBoost) as a preferred method for chicken behavior identification features. A nine-axis inertial sensor was used to obtain the chicken behavior data. After noise reduction, the sliding window was used to extract 44 dimensional features in the time domain and frequency domain. To improve the search ability of the Whale Optimization algorithm for optimal solutions, the introduction of the good point set improves population diversity and expands the search range; the introduction of adaptive weight balances the search ability of the optimal solution in the early and late stages; the introduction of dimension-by-dimension lens imaging learning based on the adaptive weight factor perturbs the optimal solution and enhances the ability to jump out of the local optimal solution. This method’s effectiveness was verified by recognizing cage breeders’ feeding and drinking behaviors. The results show that the number of feature dimensions is reduced by 72.73%. At the same time, the behavior recognition accuracy is increased by 2.41% compared with the original behavior feature dataset, which is 95.58%. Compared with other dimensionality reduction methods, the IWOA–XGBoost model proposed in this paper has the highest recognition accuracy. The dimension reduction results have a certain degree of universality for different classification algorithms. This provides a method for behavior recognition based on acceleration sensor data.

## 1. Introduction

The behavior of livestock and poultry, and, more specifically, the direct response of their physiological and psychological behavior under specific conditions, can be used as direct evidence to evaluate the welfare of breeding and chicken health. Therefore, it is of great significance to study the behavior of chickens raised in cages, and understand the production status of breeders, to improve the production efficiency of laying breeders.

An acceleration sensor is widely used in the field of behavior recognition because of its small size, high precision, stable performance, and many other advantages [1,2,3,4,5,6,7,8]. Liu Longshen et al. [9] used the logistic regression algorithm to discriminate between healthy chickens and lame chickens wearing foot rings, and used the Euclidean distance to evaluate the degree of lameness of the chickens, which was of great significance for monitoring chicken leg health and reducing economic losses. Martiskainen et al. [10] collected the acceleration data of dairy cows by fixing the sensor to the neck, and used the Support Vector Machine, or SVM, as the classification model to identify behaviors such as standing and feeding of dairy cows, although the recognition accuracy needs to be improved. Li Lihua et al. [11] used the K-means clustering algorithm to identify behaviors of chickens such as feeding and drinking. Ying Yewei et al. [12] used threshold classification and optimized SVM parameters, using a genetic algorithm, to accurately identify ewes’ feeding and drinking behaviors, which provided a reference for improving the prenatal behavior classification level of ewes. Jin Min [13] used two algorithms, ReliefF and Random Forest, to optimize the 21 dimensional features extracted from pig acceleration behavior data down to 9 dimensional features. After using a BP neural network to identify and compare the results of the two feature selections, it was found that the Random Forest algorithm effectively reduced the feature dimensions, reduced the complexity of the algorithm, and improved the recognition results. Li Xiaonan and Cheng Linglun [14,15] optimized and reduced the dimensions of the extracted acceleration features of human behavior using a genetic algorithm and ant colony algorithm, so as to realize accurate recognition of human behavior.

Feature selection is an important tool for dimensionality reduction of data, and finding optimal solutions using metaheuristic algorithms has been widely used in feature selection [16]. Some examples are Genetic Algorithm [17], Grey Wolf Optimizer [18], Artificial Bee Colony [19], Particle Swarm Optimization [20], Brain Storm Optimization [21], Gravitational Search Algorithm [22], Ant Lion Optimizer [23], and Teaching-Learning-Based Optimization [24]. The traditional metaheuristic algorithm has the problems of low convergence accuracy and slow convergence speed when dealing with complex problems. Improving the search ability of the algorithm to find the optimal solution is a hot issue in the field of the metaheuristic algorithm [25,26,27,28,29]. Xu Hui et al. [30] combined particle swarm optimization and the moth-to-flame algorithm, and they applied the improved moth-to-flame optimization algorithm to the feature selection problem of network intrusion detection. The results showed that the convergence accuracy of the improved moth-to-flame optimization algorithm was comparable to, and the speed was better than, that of the original algorithm. Zhao Zeyuan et al. [31] proposed an improved hybrid binary locust optimization feature selection algorithm. The results showed that this algorithm has a better search performance, convergence performance, and robustness. Elaziz M A et al. [32] applied the Dynamic-Opposite Learning strategy to the Atomic Orbital Search algorithm, which greatly improved the searchability of the algorithm. The Whale Optimization algorithm is a Seyedali Mirjalili [33] proposed new metaheuristic algorithm, but like other metaheuristic algorithms it also suffers from the problem of low search accuracy. In order to improve the convergence speed and global search ability of the Whale Optimization Algorithm, scholars have also carried out a lot of research, such as the introduction of a backward learning mechanism [34], the addition of the Levy flight strategy [35] and chaos mechanism [36], the introduction of adaptive weights for the algorithm [37], or the improvement of convergence factors [38]. Sayed G I [39] proposed a global optimization algorithm for feature selection based on chaos and the Whale Optimization algorithm, which introduced the regularity and semi-randomness of chaotic systems to improve the classification accuracy. Majdi Mafarja [40,41] applied the Whale Optimization algorithm to the feature selection problem and combined the Whale algorithm with the simulated annealing algorithm to improve the efficiency of classification. Yu Hang et al. [42] proposed a hybrid differential evolution algorithm based on Whale Optimization. After testing on data sets such as UCI, it was found that the accuracy of feature optimization increased significantly after dimension reduction.

There are many time-domain and frequency-domain features in the use of acceleration sensors to identify animal behavior, and the feature selection mainly depends on experience and strong subjectivity, which can mean high redundancy and affect the accuracy of model recognition. At the same time, the original Whale algorithm can easily fall into local optimization, a slow convergence speed, and low convergence accuracy. For these reasons, this paper has proposed a chicken behavior recognition feature optimization method combined with IWOA–XGBoost. The improved Whale Optimization algorithm with a mixed strategy was used to optimize and reduce the dimensions of the extracted time-domain and frequency-domain characteristics of behavioral acceleration and angular velocity. The effectiveness of this method was verified by using the recognition of feeding and drinking behavior of breeding cocks in cages as an example, providing a method for animal behavior recognition based on acceleration sensors.

## 2. Materials and Methods

### 2.1. Experimental Materials

**Figure 1:**
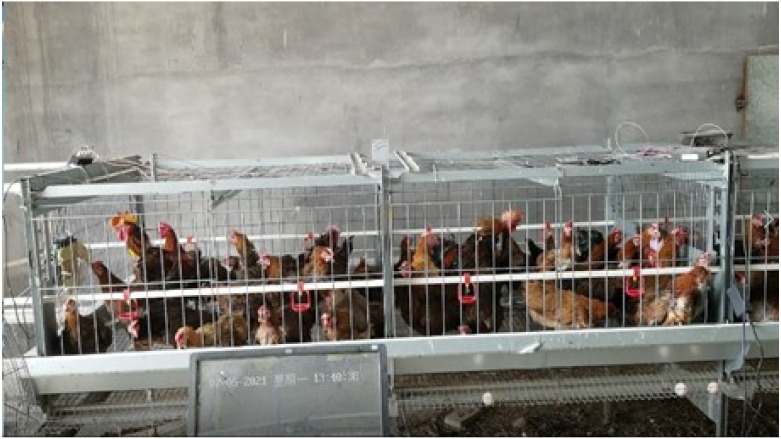
An actual picture of this cage experiment.

The relevant experiments in this paper were carried out in the Animal Husbandry Teaching Base of Hebei Agricultural University, Baoding City, Hebei Province. The size of the cage in the test base was (2400 × 1250 × 720) m^3^ and the test objects were 300-days-old Taihang chickens, a local chicken breed. Five breeding roosters and forty hens were raised in the cages at the ratio of 1:8. The experiment collected the behavior data of roosters for 7 days from 3–9 July 2021. They were fed at 9:00 a.m., 12:00 noon, and 6:00 p.m., and a nipple drinker was used to provide drinking water. The breeding site is shown in Figure 1.

### 2.2. Data Acquisition System

**Figure 2:**
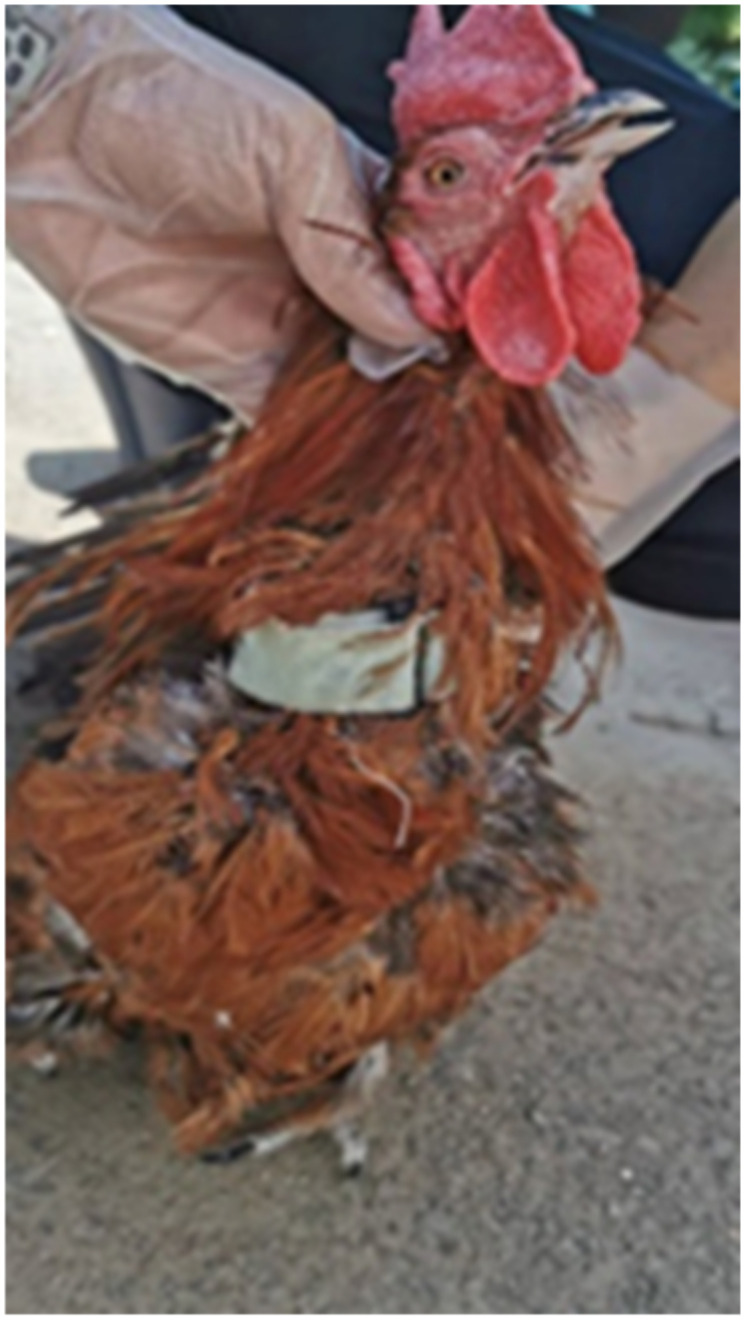
Sensor wearing mode.

In front of the chicken’s neck, we fixed a nine-axis inertial sensor model BTW901BLECL5.0 with a nylon cable tie with a self-adhesive nylon buckle, so that the X axis, Y axis, and Z axis of the sensor pointed in the outward direction, perpendicular to the chicken’s neck, in the forward direction of the chicken’s neck, and in the downward direction of the chicken’s neck, respectively. The fixing method of the sensor on the neck of the rooster is shown in Figure 2.

**Figure 3:**
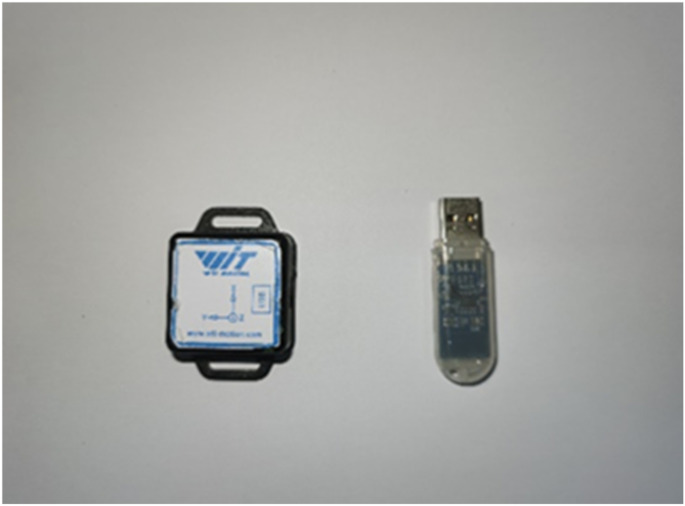
Sensor and adapter.

The size of the sensor used in this test was (51 × 36 × 15) mm^3^, the mass was 20 g, the battery life was 19 h, and the data transmission radius was able to reach 30 m. It could simultaneously collect three types of data, acceleration, angular velocity, and angle, and the collection frequency was set to 5 Hz. After the sensor collected the data, it sent the data to the multi-level connection adapter (as shown in Figure 3) through Bluetooth 5.0 and sent it to the host computer through the serial port. Each sensor number was unique and corresponded to one of the five test males. Hikvision’s network high-definition camera was installed directly above the cross cage to monitor chicken activities 24 h a day, and it was used for the identification and verification of breeding rooster behavior data.

### 2.3. Data Preprocessing

In this experiment, the acceleration sensor was used to collect the nine-axis inertial sensor and angular velocity data for the two behavioral states of feeding and drinking, and the data sets of the two behavioral states of feeding and drinking were constructed. In this study, the collected three-dimensional acceleration data were synthesized into the one-dimensional composite acceleration, and the three-dimensional angular velocity data were synthesized into the one-dimensional composite angular velocity. The calculation formula of the resultant acceleration, *a*, and the resultant angular velocity, *j*, is as follows:

(1) $$a=\sqrt{a_{x}^{2}+a_{y}^{2}+a_{z}^{2}}$$

(2) $$j=\sqrt{j_{x}^{2}+j_{y}^{2}+j_{z}^{2}}$$

where $a_{x}$, $a_{y}$, $a_{z}$, $j_{x}$, $j_{y}$, $j_{z}$ represent the acceleration and angular velocity of the *x*, *y*, and *z* axes, respectively.

When the rooster behavior information was collected by the acceleration sensor, there was noise in the data due to the installation location of the sensor and the diversity of rooster behavior. In order to improve the recognition effect, the Butterworth filter was used to denoise the resultant acceleration and angular velocity data.

The raw data of the acceleration and angular velocity could not be directly used as a sample to determine the type of behavior, and it was necessary to extract features from the data. In this paper, a sliding window was used to extract the time domain and frequency domain features of the resultant acceleration and resultant angular velocity. The time domain features that were included were the mean, number of over-mean points, upper quartile, lower quartile, interquartile difference, variance, standard deviation, maximum value, minimum value, difference between maximum and minimum values, and mode number, and the frequency domain feature was the direct current. The amplitude statistical features included were the mean, variance, standard deviation, slope, and kurtosis. The shape statistical features were the mean, variance, standard deviation, slope, and kurtosis. A total of 44 features were included overall. When the window was small, it was difficult to extract sufficient time domain and frequency domain features due to the small amount of data. In this paper, the window size was set to 14, the overlap rate was 50%, and when marking data, the data with a behavior duration of less than three seconds were excluded.

### 2.4. Behavior Recognition Methods

**Figure 4:**
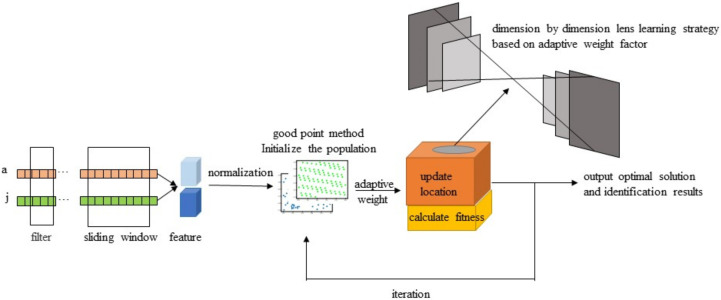
Feature selection and recognition models.

This study used python3.8 to process and analyze the acceleration and angular velocity data of the roosters’ feeding and drinking behaviors. In order to improve the recognition effect of the model, reduce the number of calculations, remove redundant features from the total feature set, and solve the feature redundancy problem in the recognition process, this paper adopted the improved Whale algorithm with the mixed strategy to reduce the feature dimensions and select the optimal feature set. The optimal recognition model of chicken behavior characteristics is shown in Figure 4.

#### Improved Whale Optimization Algorithm with Mixed Strategy

This paper proposed an improved Whale algorithm based on mixed strategies. First, we used the good point set to initialize the population to improve the diversity of the initial population. Second, we used adaptive weight factor to balance the abilities of global search and local search. Finally, based on dimension-by-dimension lens imaging learning with the introduction of adaptive weight factors, the search range was expanded and the search ability for the optimal solution was improved. In order to take into account the accuracy rate and the feature dimensions, and maximize the accuracy rate and minimize the feature dimensions, the objective function [43,44] of this paper can be defined as:

(3) $$Fitn(IWOA)=\alpha \cdot (1-Acc)+\beta \cdot (\frac{feasel}{numfea})$$

where *Acc* represents the correct rate of five-fold cross-validation for each feature combination in the XGBoost classifier, *feasel* represents the dimensions of the selected feature, and numfea represents the total dimensions of the feature. α represents the weight of classification accuracy in the fitness function, β represents the weight of the number of selected features in the fitness function, where β=1−α. The primary purpose of feature selection and dimensionality reduction is to obtain a higher recognition accuracy, where the number of features is as small as possible while ensuring a higher accuracy, so let α=0.99, β=0.01. We calculated the fitness value of each feature combination and the smaller the fitness value, the better the position.

(1) Good point set method.

**Figure 5:**
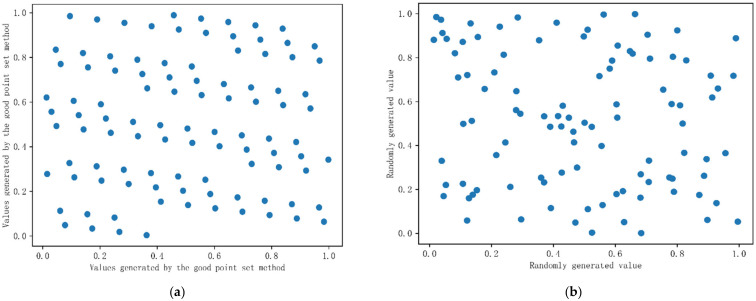
Population initialization. (**a**) The good point set method randomly generated 100 points; (**b**) the random method generated 100 points.

This paper used the method of the good point set to initialize the population. The good point set has the characteristics of uniformity and ergodicity. The points generated by the good point set in the s-dimensional space replaced the original population of the Whale algorithm in the solution space to improve the global search ability of the algorithm. A total of 100 initial populations were generated by using the good point set method and the random distribution method. There were two dimensions, let the lower limit be 0 and the upper limit be 1, and the population distribution is shown in Figure 5. It can be seen from the figure that the population distribution generated by the good point set method was more uniform, and the traversal was better. The mathematical expression of the good point set method is:

(4) $$\left\{\begin{array}{l} P_{n}(k)=\{(\{r_{1}\times k\},\{r_{2}\times k\},\cdots ,\{r_{s}\times k\}),k=1,2,\cdots N\}\\ r_{k}=2\cos(\frac{2\pi k}{q}) \end{array}\right.$$

here, r represents the good point, $P_{n}(k)$ represents the good point set, N represents the number of points, {r×k} represents the remainder, and q represents the smallest prime number satisfying (q−3)/2≥s.

(2) Adaptive weights.

**Figure 6:**
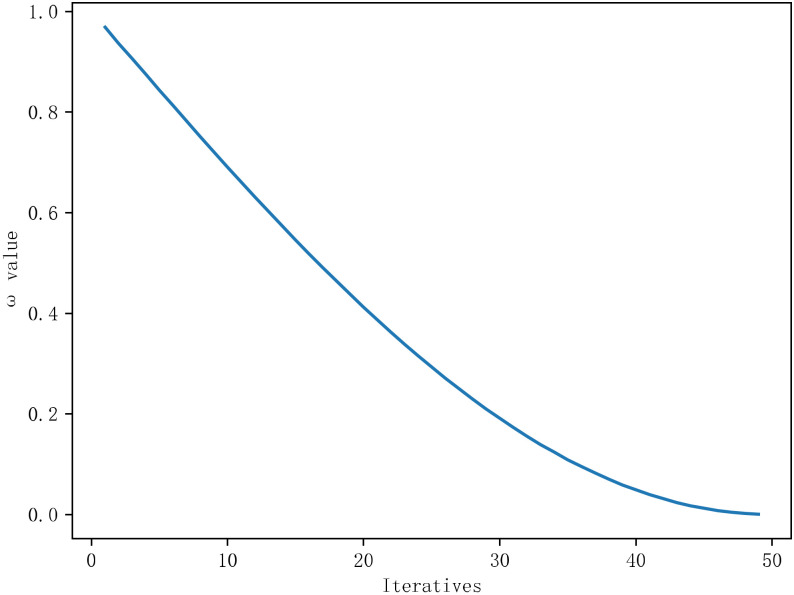
Adaptive weight factor change curve.

The adaptive weight factor ω had a larger value in the early stage of iteration, the population update step was large, and the search space was large, which was conducive to early exploration. In the later stage of the iteration, the value of ω became smaller, and the update step size of the individual population was small, which was beneficial to the development of the local search of the algorithm. The curve is shown in Figure 6. The formula for the adaptive weight factor is as follows:

(5) $$\omega =\sin\left(\frac{\pi \cdot t}{2\cdot t_{\max}}+\pi \right)+1$$

here, *t* represents the current number of iterations, and $t_{\max}$ represents the maximum number of iterations.

Then, the whale group position update method was:

(6) $$\vec{X}\left(t+1\right)=\omega \cdot \vec{X}{}^{*}\left(t\right)-\vec{A}\cdot \vec{D},\;p<0.5,\left|A\right|<1$$

(7) $$\vec{X}\left(t+1\right)=\omega \cdot {\vec{X}}_{rand}\left(t\right)-\vec{A}\cdot \vec{D},\;p<0.5,\left|A\right|\geq 1$$

(8) $$\vec{X}\left(t+1\right)=\omega \cdot \vec{X}{}^{*}(t)+\vec{D}{}^{\prime }\cdot e^{bl}\cdot \cos(2\pi l),p\geq 0.5,\left|A\right|<1$$

here, $\vec{A}$ is the coefficient vector, $\vec{D}$ is the distance between the individuals in the whale group and the current optimal position, $\vec{X}$ is the position vector of the current solution, $\vec{X}{}^{*}$ is the position vector of the current optimal solution, and ${\vec{X}}_{rand}$ is the randomly selected whale position vector. *b* is a constant that defines the shape of the logarithmic spiral, and l is a random number in the range of [−1, 1]. p is a random number in the range of [0, 1].

(3) Dimension-by-dimension lens imaging learning strategy based on the adaptive weight factor.

**Figure 7:**
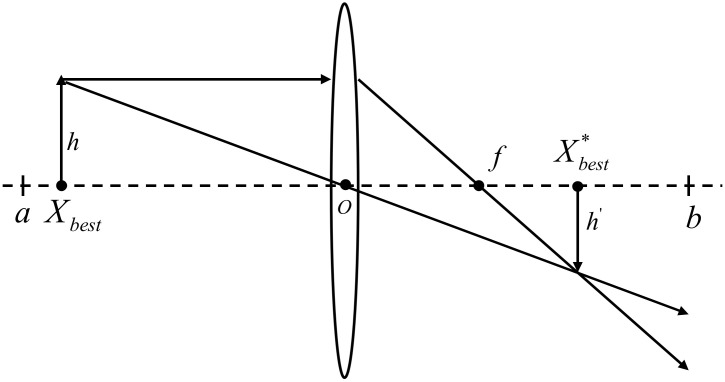
Lens learning strategy.

To address the problem that the Whale algorithm is prone to falling into the local optimum and has a poor global search ability in the later stage, this paper proposed a dimension-by-dimension lens imaging learning strategy that introduces adaptive weight factor. Assuming that the reverse point process of the whale looking for the optimal value in the solution space was similar to lens imaging, we let the current optimal solution $X_{best}$ be the projection of the individual whose height is h on the x axis, and a, b represent the upper and lower limits of the coordinate axis. Through a convex lens with a focal length f at the origin, an inverted image with a height of $h^{\prime }$ could be obtained; thus, the inverse solution $X_{best}^{*}$ of $X_{best}$ could be obtained, as shown in Figure 7. From the lens imaging principle, the following formula could be obtained:

(9) $$\frac{\left(a+b)/2-X_{best}(t\right)}{X_{best}^{*}(t)-(a+b)/2}=\frac{h}{h^{\prime }}$$

Let $\frac{h}{h^{\prime }}=n$, where *n* is the scaling factor, we can obtain:

(10) $$X_{best}^{*}(t)=\frac{\left(a+b\right)}{2}+\frac{\left(a+b\right)}{2n}-\frac{X_{best}}{n}$$

It can be seen from the above formula that when n is adjusted, the learning strategy will change to find the optimal individual, and when n=1 is a general reverse learning strategy.

In this paper, three adaptive weight factors $r_{1}$, $r_{2}$, $r_{3}$ are introduced, and combined with the lens learning strategy to mutate, a large-scale search could be performed near the optimal position in the early stage of the algorithm iteration, and a fine search could be performed near the optimal solution in the later stage to enhance the search ability of the optimal solution, which is:

(11) $$X_{best}^{*}(t)=r_{1}\frac{\left(a+b\right)}{2}+r_{2}\frac{\left(a+b\right)}{2n}-r_{3}\frac{X_{best}}{n}$$

(12) $$r_{1}=1-\log_{2}(1+\frac{t}{t_{\max}})$$

(13) $$r_{2}=1-{\left(\frac{t}{t_{\max}}\right)}^{2}$$

(14) $$r_{3}=1-{\left(\frac{t}{t_{\max}}\right)}^{3}$$

When evaluating the fitness of the original Whale Optimization algorithm, the results of all dimensions were evaluated as a whole, but better results in one dimension may have been lost. In order to fully tap the information of each dimension, the dimensional lens imaging learning strategy based on the adaptive weight factor was adopted to improve the search range.

**Figure 8:**
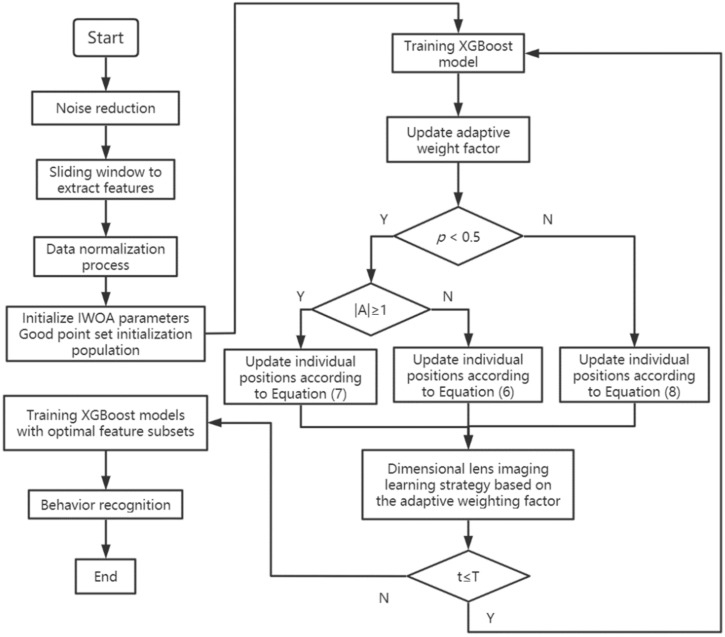
Algorithm flow chart.

In summary, the structure of the feature selection model based on IWOA for behavior identification of chickens can be obtained as shown in Figure 8.

## 3. Results

### 3.1. Noise Reduction and Feature Extraction

**Figure 9:**
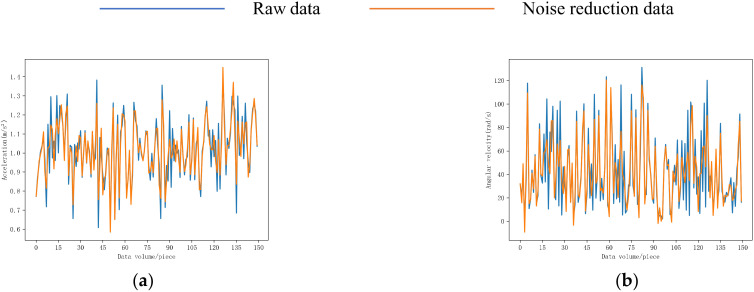

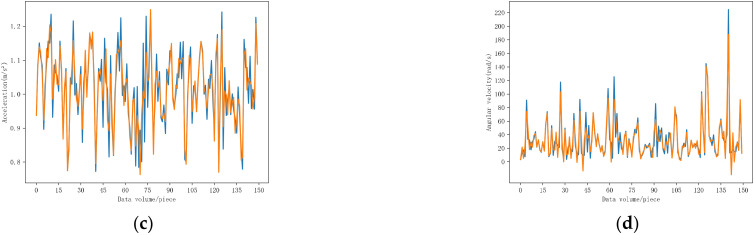
Noise reduction curve of chicken behavior. (**a**) Results of the resultant acceleration noise reduction of eating behavior; (**b**) results of denoising of the combined angular velocity for eating behavior; (**c**) results of the acceleration noise reduction of drinking behavior; (**d**) results of denoising of the combined angular velocity for drinking behavior.

The behavior data for eating food and drinking water for 30 s in this experiment were selected, and the 8-order Butterworth filter was used for noise reduction processing. The noise reduction results are shown in Figure 9. Comparing the behavior curves of the two behaviors, we can see that the fluctuation ranges of the two behaviors overlap. After filtering, the noise reduction curve became smoother than the original curve, and high-frequency noise was removed, which was beneficial to behavior recognition. A total of 1197 groups of feeding characteristic data and 779 groups of drinking water characteristic data were extracted through the sliding window.

### 3.2. Comparison of the IWOA–XGBoost and WOA–XGBoost Models

**Figure 10:**
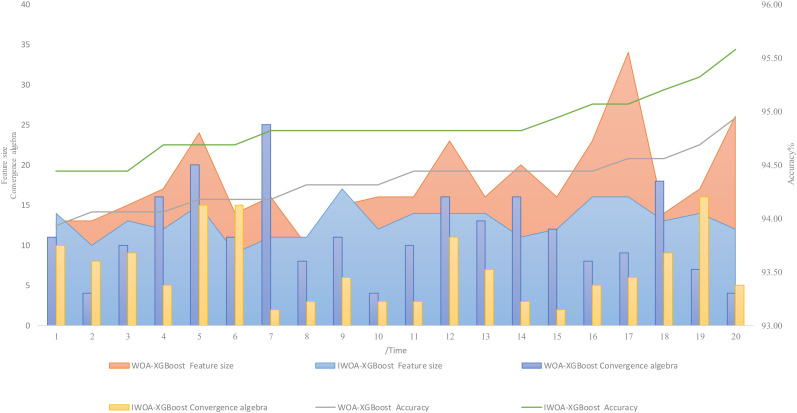
Comparison of the WOA–XGBoost and IWOA–XGBoost algorithms run 20 times.

We set the population size of the WOA and IWOA optimization algorithms to 15 and the maximum number of iterations to 50. The data were randomly divided into the training set and test set at a ratio of 6:4 and were normalized, then run 20 times under the same hardware conditions. The model accuracy, fitness value, convergence algebra, and feature size are shown in Table 1. When the IWOA–XGBoost algorithm performed feature selection, the highest recognition accuracy was 95.58%, the lowest recognition accuracy was 94.44%, and the average recognition accuracy was 94.81%, which were 0.64%, 0.51%, and 0.51% higher than the WOA–XGBoost algorithm, respectively. The maximum fitness value was 0.0583, the minimum fitness value was 0.0465, and the average fitness value was 0.0539, which were 0.0047, 0.0095, and 0.0061 lower than the WOA–XGBoost algorithm. The average convergence algebra was 7.15, which was 4.5 lower than the WOA–XGBoost algorithm. The average number of feature dimensions was 13, which was 4.9 lower than the WOA–XGBoost algorithm. The WOA–XGBoost and IWOA–XGBoost algorithms were run twenty times each, and the distributions are shown in Table 1. The results are shown arranged according to the accuracy rate from small to large, the accuracy rate, convergence algebra, and feature size of each running result in Figure 10. It can be seen that the improved Whale algorithm with the hybrid strategy constructed in this paper optimized the behavior of breeders for feeding and drinking. The performance of dimensionality reduction was obviously better than the original algorithm in terms of convergence speed, recognition accuracy, and feature dimensions.

### 3.3. Feature Dimensionality Reduction Effect

In order to compare the effect of feature optimization and dimension reduction on the accuracy of model identification of chicken behavior, the original feature set and optimal feature subset were identified using the XGBoost model. The recognition result of the feature combinations with the highest accuracy was taken as the optimal feature combination, including seven acceleration features, which were the lower quartile (f1), upper quartile (f2), variance (f3), and maximum value (f4); the shape statistical features were the mean (f5) and standard deviation (f6); and the amplitude statistic was kurtosis (f7). There were five angular velocity features, which were the number of over-average points (f8) and direct current (f9); the shape statistical features were the mean (f10) and slope (f11); and the amplitude statistic was the mean (f12). The experimental recognition results before and after feature reduction are shown in Table 2. The recognition effect of the model was significantly improved after feature dimensionality reduction, 458 eating behaviors and 298 drinking behaviors were correctly recognized, in which the accuracy of foraging behavior recognition was improved from 94.00% to 97.03%, an improvement of 3.03%, the recall of drinking behavior was improved from 90.71% to 95.51%, an improvement of 4.8%, the model accuracy was improved by 2.29% to 95.23%, model recall improved by 2.82% to 95.57%, model F1 score improved by 2.54% to 95.39%, and model accuracy improved by 2.41% to 95.58%. In terms of feature dimensions, the IWOA–XGBoost model established in this paper reduced 44 dimensional features to 12 dimensions, and the feature dimensions were reduced by 72.73%, removing a large number of redundant features and reducing the computation requirements. The recognition accuracy of this model was high, which would enable it to meet the need for accurate recognition of chicken feeding and drinking behavior.

**Figure 11:**
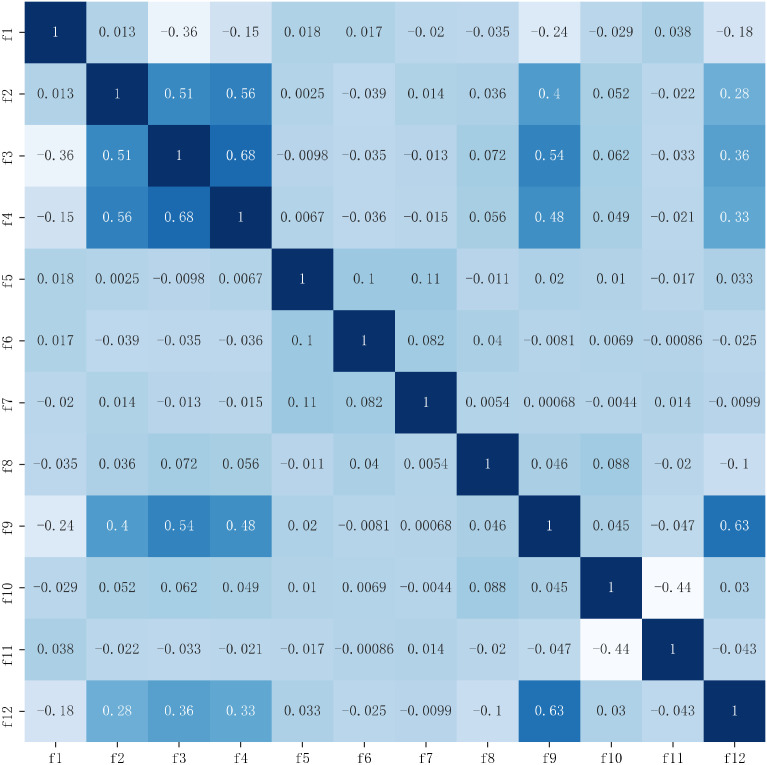
Kendall coefficient matrix.

**Figure 12:**
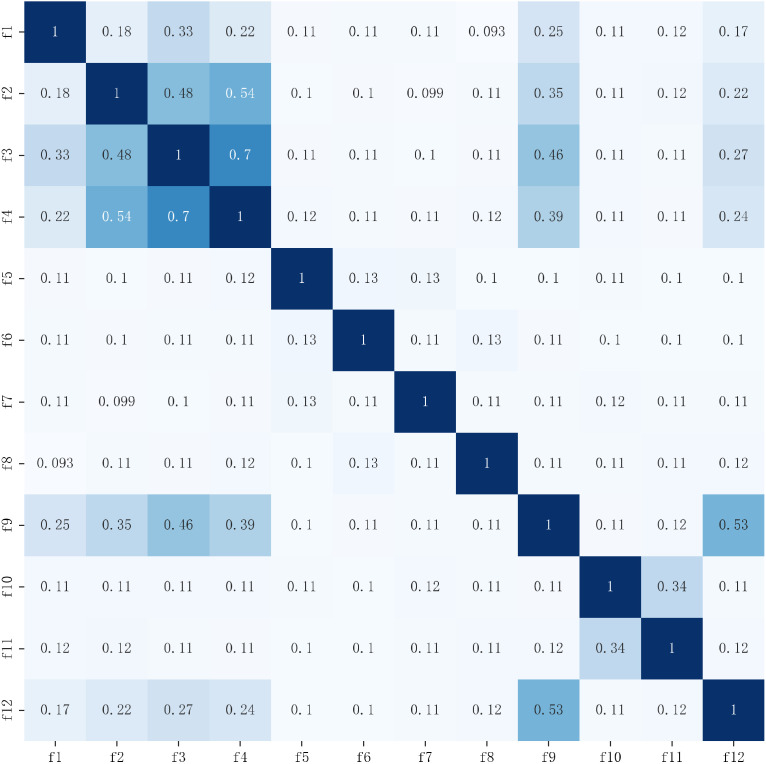
MIC coefficient matrix.

The Kendall correlation coefficient and the Maximal Information Coefficient (MIC) were used to evaluate the dimensionality reduction effect. The Kendall correlation coefficient can measure the dependence of the two variables, and the Maximum Mutual Information Coefficient measures the amount of information contained in the two variables. The coefficient matrix is shown in Figure 11 and Figure 12. When the Kendall correlation coefficient and MIC coefficient are 0, it means the two features are completely independent of each other, and when the coefficient value is 1, it means the two features are completely correlated, where a correlation coefficient greater than 0.8 is usually defined as a high correlation. The Kendall correlation coefficients are greater than 0.6 for the variance (f3) and maximum value (f4) in acceleration and the direct current (f9) and the mean of amplitude statistic (f12) in angular velocity. The MIC coefficients are greater than 0.6 for the variance (f3) and maximum value (f4). The coefficients did not exceed 0.7 [45], which is a medium strength correlation, while the correlations for the remaining features are weak. To further explore whether there is a fully substitutable relationship for features with high relevance, features are removed one by one to observe the effect on recognition accuracy. Removing the variance and the maximum value, the direct current, and the mean of amplitude statistics, respectively, means that the recognition accuracy of the XGBoost classification model was 92.54%, 94.44%, 94.44%, and 94.46%, which were all less than 95.58% of the optimal feature combination. It shows that although there is a medium strength correlation between features, for variables with high correlation, neither variable can fully explain the information of another variable, and the IWOA–XGBoost model established in this paper is based on recognition accuracy as the evaluation criterion. Therefore, the variables with medium strength correlation are retained to improve the accuracy. So, the IWOA–XGBoost model established in this paper can maintain a good recognition effect while reducing dimensionality and redundancy.

To sum up, the IWOA–XGBoost feature optimization dimensionality reduction model established in this paper can remove irrelevant features and weakly related features that have little or a negative impact on the model recognition results when faced with features with high dimensionality. Strong correlation features that play a positive role in the recognition results can reduce the feature dimensions and reduce the number of calculations, while improving the accuracy of model recognition.

### 3.4. Feature Importance Analysis

**Figure 13:**
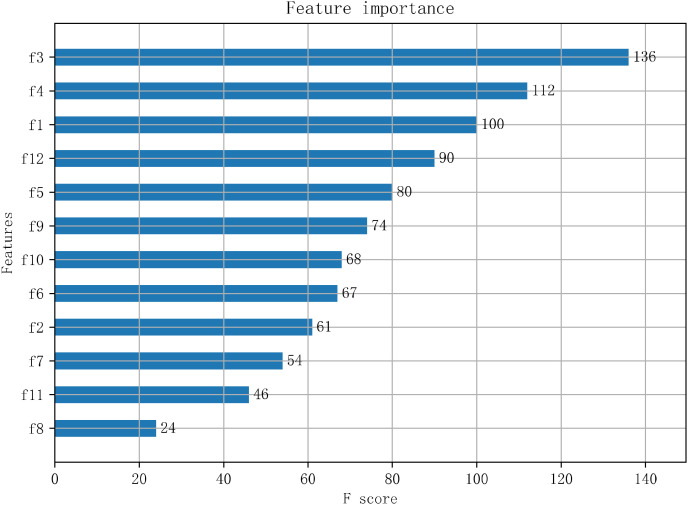
Feature importance measure F-score value.

Using the XGBoost algorithm to measure the feature importance of the 12 features after the optimization of the dimension reduction, the F-score value is shown in Figure 13. Among them, the variance, the maximum value, and the lower quartile in the acceleration feature are of high importance. The variance represents the degree of dispersion and fluctuation of the acceleration data. The greater the variance, the greater the fluctuation of the data and the greater the change in behavior. The maximum value is an important feature to describe the intensity of data changes. The larger the maximum value, the greater the range of motion of the behavior, and the more obvious the fluctuation of the behavior curve.

### 3.5. Algorithm Performance Comparison

**Figure 14:**
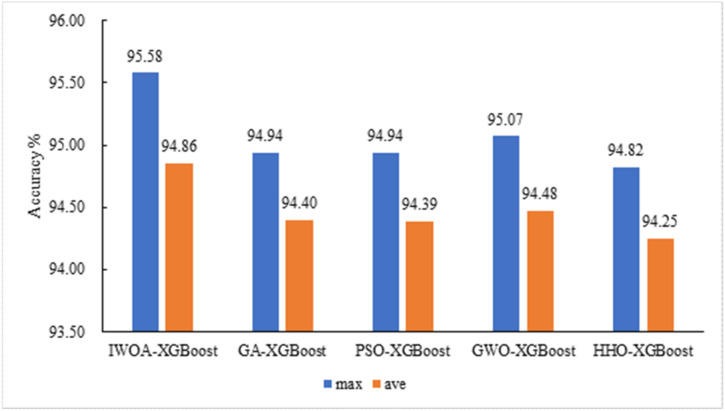
Comparison of dimensionality reduction results of different algorithms.

In order to verify the superiority of the IWOA–XGBoost algorithm proposed in this paper, Genetic Algorithm (GA), Grey Wolf Optimization (GWO), Particle Swarm Optimization (PSO), and Harris Hawks Optimization (HHO) are selected under the same hardware conditions, and the population size is set to 15, the maximum number of iterations is 50, and the original feature set is selected for feature identification. After running 20 times, the maximum and average values of the recognition accuracy are shown in Figure 14. It can be seen from the figure that the IWOA–XGBoost model constructed in this paper has the highest recognition accuracy, and the average accuracy is also at the highest level.

### 3.6. Comparison of Universality of Feature Subsets

In order to verify the effectiveness of the IWOA–XGBoost method adopted in this paper, under the same computer and software platform environment, the four models of Logistic Regression, Decision Tree, GaussianNB, and LightGBM were used to identify the behavior data samples before and after feature optimization. The recognition results of the four classification algorithms are shown in Table 3.

It can be seen from the table that after feature optimization, the four models had improved recognition accuracy of feeding and drinking behaviors of breeders to varying degrees, indicating that the IWOA–XGBoost feature optimization model constructed in this paper has a certain universality. By comparison, it was found that the IWOA–XGBoost model had a higher recognition accuracy, precision, recall, and F1 score, and can achieve accurate identification of feeding and drinking behaviors of cross-cage breeders.

## 4. Discussion

The acceleration and angular velocity data of chicken behavior include rich information. Statistical features such as the time domain and frequency domain are included in the comprehensive manifestation of behavior data. The extraction and selection of data features is an important factor affecting recognition results. This paper proposed a feature optimization method for chicken behavior recognition based on nine-axis inertial sensor data and used a hybrid strategy improved Whale Optimization algorithm to effectively solve the feature redundancy problem in the behavior recognition process.

From the point of view of the feature optimization model, the behavioral features of chickens in this paper had as many as 44 dimensions. With the increase of feature dimensions, the number of feature subsets increases exponentially. Using all features for classification will greatly increase the time and computational complexity of the model. The introduction of behavioral features will also affect the final classification effect. In this paper, when the original Whale Optimization algorithm was used to reduce the dimensions, the best recognition effect was achieved when there were 26 feature dimensions. The improved Whale Optimization algorithm with a hybrid strategy improved the search ability of the optimal solution, and the highest accuracy rate was increased by 0.64%. At the same time, there were only 12 feature dimensions, which greatly improved the performance of the model.

From the point of view of feature set selection, the selection of the traditional behavior recognition feature set depends on previous experience or the relationship between the mathematical meaning of the feature and the behavior, but for different recognition objects, the selection of strong correlation features to be retained is different from the redundant features to be eliminated, which has a greater impact on the recognition results [46,47]. The optimal feature subset obtained in this paper for identifying the feeding and drinking behavior of the cross-cage breeders effectively represented the behavior information of the chickens.

From the perspective of dimensionality reduction universality, the optimal feature subsets screened in this paper improved the chicken behavior recognition accuracy of the logistic regression, decision tree, Naive Bayes, and lightweight gradient boosting machine models, indicating that redundant features affect the recognition effect of classifiers. This is ubiquitous, and the feature optimization method proposed in this paper can not only improve the recognition effect of XGBoost, but also improve the performance of models such as logistic regression.

There are still shortcomings in this study. The dimension-by-dimension lens imaging learning strategy that introduces adaptive weight factors has the problem of requiring a large number of calculations due to the disturbance from each dimension, and the acceleration and angular velocity information of the X, Y, and Z axis were not extracted in the study. If all feature dimensions were extracted, there would be as many as 176 dimensions, which would require a higher search ability of the model for the optimal feature set. In the follow-up research, the global search ability of the algorithm will be further improved, and chicken behavior information will be enriched. The research scope will be expanded, and the different behaviors of chickens will be more accurately identified.

## 5. Conclusions

In this study, based on the chicken behavior recognition data collected by the nine-axis inertial sensor, a behavior recognition feature optimization method based on the improved Whale Optimization algorithm with mixed strategy (IWOA) and the extreme gradient boosting algorithm (XGBoost) based on the hybrid strategy was proposed. The main conclusions are as follows:

(1) The hybrid strategy of a good point set, adaptive weight, and dimension-by-dimension lens imaging learning based on the adaptive weight factor proposed in this paper can improve the convergence speed of the original Whale Optimization algorithm and the recognition accuracy of the classification model. The average convergence algebra was reduced by 4.5, the recognition accuracy was improved by 2.41% after the feature dimensions were reduced by 72.73%, and the average precision, average recall, and average F1 score were improved by 2.29%, 2.82%, and 2.55%, respectively.

(2) The selection of different feature sets has a greater impact on the behavior recognition results. The 12 dimensional feature combination of combined acceleration and combined angular velocity screened out by the feature optimization method proposed in this paper was able to fully reflect the feeding and drinking behavior information of the chickens. The three features of variance (f3), maximum value (f4), and lower quartile (f1) were highly important. The variance (f3) and maximum value (f4) in acceleration and the direct current (f9) and the mean of amplitude statistic (f12) in angular velocity were of medium strength correlation, but they were all positive for model recognition.

(3) The four classification models of logistic regression, decision tree, Naive Bayes, and LGBM were used to identify the behavior of the feature subsets before and after optimization, and the recognition accuracy was increased by 0.71%, 1.13%, 0.88%, and 0.76%, respectively, indicating that the feature optimization method has a certain universality for different classification algorithms.

## Figures and Tables

**Table 1:** Comparison of the WOA–XGBoost and IWOA–XGBoost algorithms.

Method	Distribution	Accuracy %	Fitness Value	Convergent Algebra	Feature Size
IWOA–XGBoost	max	95.58	0.0583	16	24
	min	94.44	0.0465	2	9
	ave	94.86	0.0539	7.15	13
WOA–XGBoost	max	94.94	0.063	25	34
	min	93.93	0.056	4	10
	ave	94.35	0.060	11.65	17.9

**Table 2:** Results of chicken behavior recognition before and after feature selection.

Method	Ture Behavior	Predicted Behavior		Total	Precision %	Recall %	F1 Score %	Accuracy %
		Eating	Drinking					
IWOA–XGBoost	Eating	458	21	479	97.03	95.62	96.32	95.58
	Drinking	14	298	312	93.42	95.51	94.45	95.58
	total	472	319	791	95.23	95.57	95.39	95.58
XGBoost	Eating	454	25	479	94.00	94.78	94.39	93.17
	Drinking	29	283	312	91.88	90.71	91.29	93.17
	Total	483	308	791	92.94	92.75	92.84	93.17

**Table 3:** Comparison of the recognition results of four classification algorithms.

		Before Feature Optimization				After Feature Optimization			
Method	Behavior	Precision	Recall	F1-Score	Accuracy	Precision	Recall	F1-Score	Accuracy
Logistic Regression	eating	94.21	95.2	94.7	93.35	95.00	95.2	95.1	94.06
	drinking	92.51	91.03	91.76		92.60	92.31	92.46	
Decision Tree	eating	91.24	93.53	92.37	90.64	94.42	91.86	93.12	91.78
	drinking	89.67	86.22	87.91		88.00	91.67	89.80	
GaussianNB	eating	94.69	93.11	93.89	92.67	94.58	94.78	94.68	93.55
	drinking	89.69	91.99	90.82		91.96	91.67	91.81	
LightGBM	eating	95.38	94.78	95.08	94.06	95.82	95.62	95.72	94.82
	drinking	92.06	92.95	92.5		93.29	93.59	93.44	

## Data Availability

Not applicable.
